# Factitious Disorder as a Skin Ulcer: A Case Report

**DOI:** 10.7759/cureus.51642

**Published:** 2024-01-04

**Authors:** Konstantinos Kontoangelos, Fiori Kousta, Irene Potouridou, Vasiliki Chasapi, Dimitris Dikeos, Alexander Stratigos

**Affiliations:** 1 1st Department of Psychiatry, Eginition Hospital, National and Kapodistrian University of Athens, Athens, GRC; 2 Department of Dermatology and Venereology, Andreas Syggros Hospital, Athens, GRC; 3 1st Department of Dermatology, Hospital of Venereal & Dermatological Diseases, National and Kapodistrian University of Athens, Athens, GRC

**Keywords:** ankle, psychodermatology, ulcer, skin, psychosomatic, depression, factitious disorder

## Abstract

Dermatitis artefacta (factitious dermatitis) is a dermatological disease of different types; it could appear on various parts of the body. It is associated with severe difficulties, such as psychic distress and negative feelings aroused in healthcare personnel or borderline personality disorder, and the long-term possibility of patient self-harm to create more symptoms, resulting in unnecessary medical procedures.

This is a case of a 17-year-old girl who was hospitalized with a skin ulcer on her right ankle that proved to be a factitious disorder. She was experiencing severe symptoms of anxiety, such as feeling nervous, having trouble sleeping and concentrating, and an inability to control worry due to her preparation for university studies. She refused to see a mental health professional since the onset of anxiety symptoms, i.e., the last four months.

Patients who present with factitious disorder deliberately create clinical signs of a somatic disease because they need warmth and attention in a medical environment. Symptoms offer no significant benefit, and the pathophysiological mechanisms are mainly psychological. The primary treatment for factitious disorder is psychotherapy while the management of the ulcer requires dermatosurgical treatment.

## Introduction

The skin, the largest organ in the body, is derived from the embryonic neuroectoderm and develops a two-way physiologic dialogue between central nervous system functioning and emotional reaction along with the cutaneous expression [1]. Dermatitis artefacta or factitious dermatitis is a psychocutaneous disorder in which patients consciously create lesions in skin, hair, nail, or mucosae to satisfy a psychological need, attract attention, or evade responsibility, and this is associated with the female gender [2]. Patients are remarkably unconcerned about lesions that seemingly should be painful and certainly are disfiguring. The patients are also angry and resentful and seem to suggest that the medical community is uncaring and incompetent [3,4]. Clinical symptoms in patients with factitious dermatological disorder show great diversity and are connected with the way that the patients traumatize their skin. The skin lesions may present as erosions, ulcers, eschars, blisters, or nodules after injections [5]. Lesions are more obvious on the face, legs, and arms, and can also occur in children and adolescents; the main motivation is the assumption of the sick role of the patient with surreptitious actions to misrepresent, simulate, or cause signs or symptoms of illness in the absence of obvious external rewards and due to an unconscious psychological need [6,7]. Typically, there is a predominance related to a mental illness or psychological disorder caused by stress at work, family problems, or bereavement, and it can symbolize a distressed expression of the need for support and help [8]. This is a case report of a 17-year-old girl who was hospitalized due to a skin ulcer on the right ankle. During the clinical investigation, it was revealed that the skin lesion was caused by the patient herself.

## Case presentation

**Figure 1:**
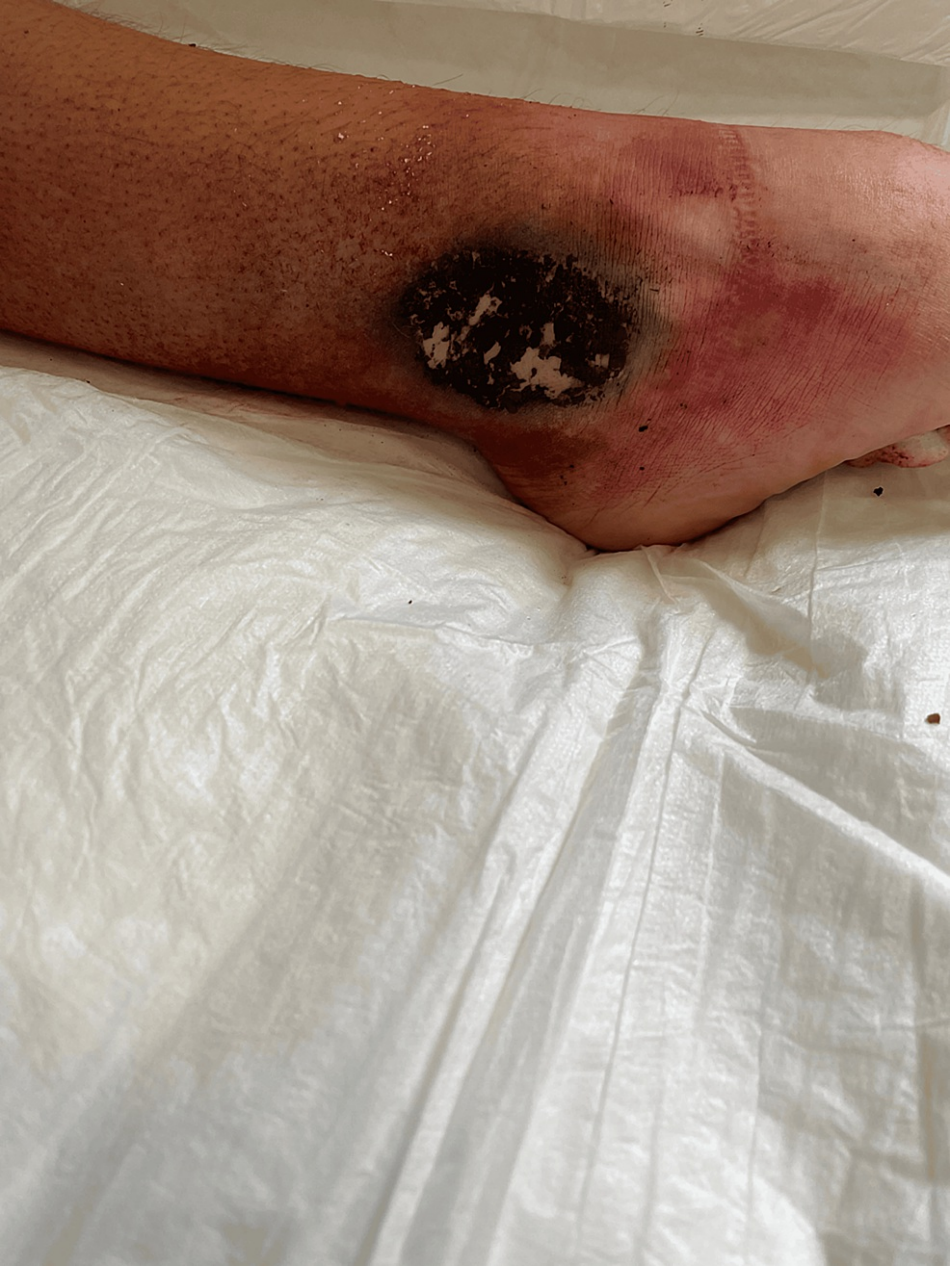
Skin lesion of the ankle presenting as an ulcer

Here, we present a clinical occurrence of a 17-year-old girl who urgently visited the outpatient clinic of the dermatology clinic at a teaching hospital, accompanied by her mother. She reported a 10-day-old skin ulcer, which began to develop on the medial side of the left leg at the ankle. According to the clinical assessment, the skin lesion started to show small redness around the ankle skin, which was black in the center presenting the appearance of an ulcer with a necrotic center and erythematous borders, affecting just the top layers of the skin (the epidermis) (Figure 1).

**Figure 2:**
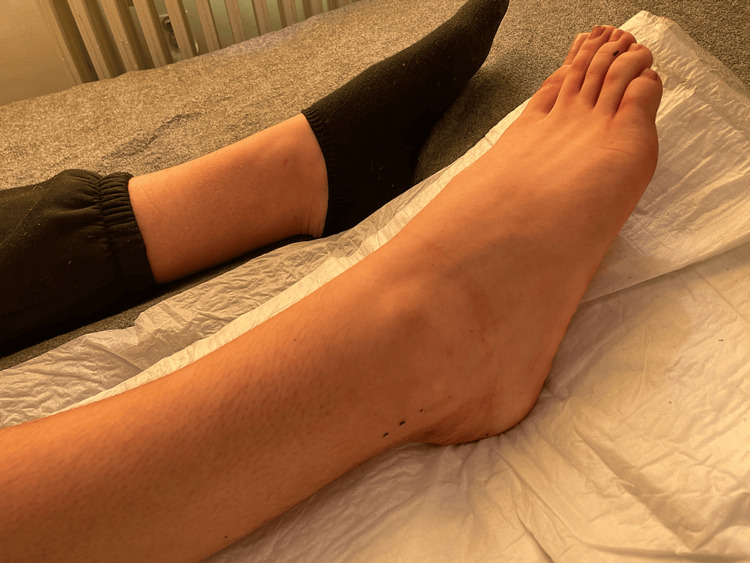
Skin lesion disappeared after disinfection of the ankle

She reported pain and skin sensitivity in the area, which made it difficult for her to attend classes, so she stopped going to school. Her mother was worried and told the emergency doctors that she had not realized the seriousness of the situation and that her daughter had only informed her a day previously; the family doctor recommended an immediate visit to a dermatology clinic. The patient was immediately admitted to the hospital for laboratory and clinical control. Blood tests and X-rays were done and an electrocardiogram was prepared. During the preparation and placement of the electrodes on the legs, and while the doctor disinfected the ankle with water, the skin lesion disappeared immediately and the ‘ulcer’ was completely washed away by water (Figure 2). At that point, she got embarrassed and burst into tears.

The dermatologist called the hospital's psychiatrist, and a psychiatric assessment was made because the specific skin damage turned out to have been caused by herself. She spoke openly about her anxieties. She revealed that she was unbearably stressed by school and everyday life, as well as the preparation for the exams and university admission, which she strongly desired. In the last two months, she had often had palpitations, sweating, chest pain, dizziness, and chills or heat sensations. Most of the day, her mood was depressive with insomnia, fatigue, and a feeling of worthlessness, in addition to a diminished ability to think or to concentrate. She was suffering from depression with feelings of sadness, hopelessness, and reduced energy. The symptoms of anxiety also intensively created feelings of nervousness, worry, or dread.

During the interview, the topic of skin damage was discussed. She admitted that she caused the skin damage with a marker to provoke the mother's sympathy because she was pressuring her to study. She had never visited a mental health specialist. She was referred for regular psychiatric follow-up and psychotherapeutic support to the mental health center of the city she came from.

## Discussion

In factitious disorder, the main clinical symptom is the purposeful creation and dissimulation of physical and psychological features to play the role of the patient [9]. Despite the fact that factitious activities are consciously formed, subjacent incentives are determined to be insensitive, and, in most cases, the main characteristics are a connection with the medical system and poor, maladaptive coping skills. Patients with factitious illness consciously produce the signs or symptoms of psychical illness although their motivation for doing so is primarily unconscious, which is also related to the psychological state of the patient [10].

Patients frequently present immature behavior regarding interpersonal skills and do not meet the criteria for any specific category of personality disorders but can relate to psychological factors such as longing for nurturance, feelings of helplessness, desire to feel superior, and anger toward physicians [11]. Documented triggering events in children and adolescents include stress from exams, parental divorce, sexual abuse, personality disorders, anxiety, depression, and emotional neglect, which may also result in response to stressful conditions, and they are frequently found in association with PTSD [12,13]. A similar stress situation affected the patient who presented with the anxiety of performing in the exams but also the fear of comparison since her older sister managed to succeed in the exams.

Feily et al. presented a case of ulceration on the dorsal surface of the glans penis in a 49-year-old man, resembling a skin lesion with an ulcer. A diagnosis of dermatitis artefacta was confirmed due to the strange and rare findings and the absence of findings from the clinical examinations. A referral was made for a neuropsychological examination [14].

Another example that supports the case presented is the clinical case of a 60-year-old married woman, the clinical assessment showed quite different shapes and well-defined ulcers on the skin of the left leg, mainly on the anterior surface of the shin. Based on the history given by the patient, the skin lesions appeared suddenly. The patient considered the appearance of the needles as the result of witchcraft [15].

Similarly, in another case, a 31-year-old woman presented with a history of recurrent skin and oral lesions for several years. In a histopathological report presented by her, she confirmed the diagnosis of pemphigus vulgaris (PV), which turned out to be false. Clinical dermatological assessment of the skin and mouth revealed multiple linear erosions on the upper lip. The psychiatric assessment established a diagnosis of borderline personality disorder, which made it difficult for the patient to manage her grief over the death of her parents [16].

In our female patient, playing the role of the patient may be considered "a cry for help" for the difficult everyday life she experienced due to the stress she felt regarding her attendance and performance at school, the absence of the father because the parents were separated and he lived far away without frequent communication with the patient, and the cold and competitive relationship with the mother. Her behavior is supported by the literature in that patients with factitious disorder are very resistant to any suggestion that their skin lesions may be self-induced. She calmly accepted admission to the hospital with the main motive of earning the family's care, ignoring the possibility that the deception would be revealed, thereby exposing herself irreparably.

Although the disorder is more associated with physical illnesses, some patients suffer from mental illnesses, but in these cases, the diagnosis is difficult. Patients with these symptoms do not often visit psychiatrists because of the low esteem they have for psychiatry and the fear they have of not revealing their true motives [17].

Antidepressants or antipsychotic medication can help, but in some cases, it is unnecessary. Psychiatric referrals of these patients should be evaluated with care because they may interpret this referral as rejection, and this may exacerbate the self-injurious behaviors on the skin. Follow-up studies have shown that patients with factitious disorder may display improvement because of changes in the conditions in their lives and the maturation of age, and less as a psychotherapeutic achievement [18]. One survey reported a prevalence of 4% in 457 institutionalized children and adolescents with mental retardation. It is essential to adopt a supportive and nonjudgmental approach to treat this condition. Relaxation exercises, anxiolytics, antidepressants, and low-dose second-generation antipsychotics are known to be helpful. Antidepressants can be useful in the presence of depressive symptoms. Selective serotonin reuptake inhibitors (SSRIs) have a better acceptance, with fewer adverse effects. However, they should be administered in the upper dose range to achieve therapeutic response. In some cases, antipsychotics may be useful, with low-dose atypical antipsychotic agents being the treatment of choice. Antipsychotics may be particularly effective in patients with the delusional type of diagnostic assessment (DA) [19].

Factitious disorder causes disability to the patient, often resulting in serious injury or adverse reactions related to the treatment [9]. A favorable prognosis is determined by the absence of antisocial personality disorder and by having a work-life balance that is found to be borderline and not at a continuous psychotic level. The treatment focuses more on the management and an important factor is the early recognition of this disorder by the family doctor. Working psychotherapeutically with the patient's physician is more effective than working with the patient alone [20].

## Conclusions

In dermatologic practice, the differential diagnosis in situations that have atypical presentations is significant and should be questioned thoroughly. The differential diagnoses to be considered for crusted, blistering lesions include ecthyma and herpes simplex. Others may simulate porphyria cutanea tarda, epidermolysis bullosa acquisita, amyloidosis, vasculitis, pyoderma gangrenosum, cutaneous lymphoma, and drug eruption. Hospital doctors must be alert for this specific diagnosis to recognize patients with factitious disorder and a certain type of psychotherapy is essential (e.g. psychoanalytic psychotherapy) in the case of the patient presented.

Patients who end up with this disorder have emotional needs and have severe difficulty communicating with other humans because of this, and the lack of human contact and warmth is masked by illness and medical symptoms. The clinician may suspect factitious disorder when there are indications that the symptoms are not true and when the presence of a benefit is discernible. The psychotherapeutic approach in collaboration with the patient's doctor is more effective than working with the patient alone.
